# Erxian herbal pair enhances bone formation in infected bone nonunion models and attenuates lipopolysaccharide-induced osteoblastinhibition by regulating miRNA-34a-5p

**DOI:** 10.1080/21655979.2022.2085388

**Published:** 2023-01-24

**Authors:** Li Zhang, Yang Zhang, Maomao Miao, Shaoqi Hu, Xuping Wang, Lisha Zhao, Xiaowen Huang, Gang Cao, Dan Shou

**Affiliations:** a School of Pharmaceutical Sciences, Zhejiang Chinese Medical University, Hangzhou 310053, China; b Institute of Orthopadics and Traumatology, the First Affiliated Hospital of Zhejiang Chinese Medical University, Hangzhou 310053,China; c Department of Medicine, Zhejiang Academy of Traditional Chinese Medicine, Hangzhou 310007, China; d Department of Pharmacy, The First Affiliated Hospital of Zhejiang Chinese Medical University (Zhejiang Provincial Hospital of Traditional Chinese Medicine), Hangzhou 310006, China

**Keywords:** Erxian herbal pair, bone repair, LPS-induced bone loss, MiRNA-34a-5p, osteoblasts

## Abstract

Bacterium-induced inflammatory responses cause bone nonunion. Although antibiotics suppress infection, bone loss after antibacterial treatment remains a critical challenge. Erxian herbal pair (EHP) has been proven effective in promoting bone formation. Our study aimed to investigate the effect of EHP on bone repair after anti-infection treatment, explore its effect on a lipopolysaccharide (LPS)-induced osteoblast. We evaluated effects of EHP on bone repair with Micro-CT, and morphology detecting. Chemical constituents of EHP and EHP-containing serum (EHP-CS) were identified by UHPLC-Q/TOF-MS. In addition, osteoblast induced by LPS was established and administrated with EHP-CS. Cell proliferationwas assessed by MTT. *Target prediction identified SMAD2 as a* *potential target of miRNA-34a-5p.* MiRNA mimic, inhibitor and siRNA were transiently transfected into osteoblasts. The mRNA levels and protein expressions of miRNA-34a-5p, BMP2, Runx2, SMAD2 were assessed. The results showed that the main biocactivity ingredients in EHP-CS were Baohuoside Ι and Orcinol Glucoside. EHP could promote bone remolding after anti-infection therapy and restore the activity of LPS-induced osteoblasts. Moreover, miRNA-34a-5p was dramatically downregulated and SMAD2 was upregulated after LPS stimulation, while EHP resisted the inhibition of LPS by promoting miRNA-34a-5p, ALP, and BMP2 expressions. Whereas downregulation of miRNA-34a-5p reversed these effects. Silencing endogenous SMAD2 expression markedly promoted BMP2 and ALP activity and enhanced osteogenesis. Taken together, EHP restored LPS-induced bone loss by regulating miRNA-34a-5p levels and repressing its target gene SMAD2. EHP might be a potential adjuvant herbal remedy for the treatment of bone nonunion, and miRNA-34a-5p is a novel target for controlling bone and metabolic diseases.

## Graphical abstract

**Figure:**
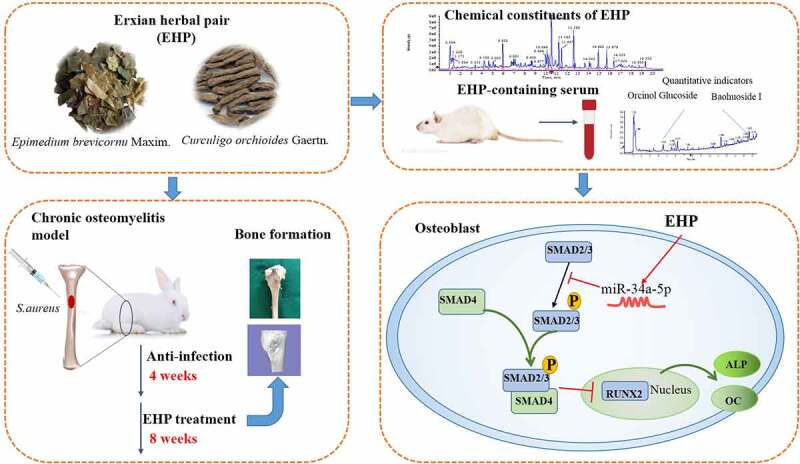

## Highlights

- EHP could promoted bone remolding after anti-infection therapy.
- The main biocactivity ingretients in EHP-CS were Baohuoside Ι and Orcinol Glucoside.
- EHP protected LPS-induced osteoblast.
- EHP resisted inhibitory action of LPS on osteoblast by regulating miR-34a-5p, and BMP2/SMAD2/Runx2 pathway.

## Introduction

Infectious bone disease is mainly caused by bacterium invading and characterized by inflammation-associated bone defect with concurrent new bone formation [1]. There are approximately 10,000 fracture patients in China each year, and the incidence of nonunion is approximately 5%-10%, most cases of which are caused by open fracture and traffic accidents inducing infection, as well as the regularity use of orthopedic devices. The predominant therapies for infectious bone disease are surgery to thoroughly remove dead bones and inflammatory granulation tissue and to provide local and systemic application of sensitive antibiotics to control infection [2]. With the widespread application of antibiotics, many pathogenic bacteria have developed resistance, which is difficult to treat owing to its higher risk of infection recurrence. Recent treatments require complicated operations; however result in long-term disablement, contributing to high costs for the patients [3]. Currently, infected bone nonunion remains one of the most challenging disorders for orthopedic surgeons. Therefore, there is a need to seek effective treatments for infected bone nonunion.

Previous resarches have suggested that natural herbal products have shown promising potential for enhancing bone formation [4]. EHP, a traditional Chinese herbal pair that includes *Epimediumbrevicornu* Maxim. and *Curculigo orchioides* Gaertn., has been widespread for treating bone metabolism diseases, such as osteoporosis, osteoarthritis, and chronic osteomyelitis [5,6]. EHP has been reported to display inhibitory effects on osteoclastic bone resorption and positive effects on osteoblast proliferation [7]. Recently, Wong [8] et al. reported that EHP had a definite anti-osteoporotic effect similar to estrogen. Zhu [9] et al. reported that EHP promotes osteoblasts proliferation, and inhibits apoptosis. However, the accurate mechanism for mediating osteoblast differentiation and bone repair has not yet been studied.

MicroRNAs (miRNA) are small noncoding RNAs, and have been proved to regulate gene expression by binding to 3'-untranslated regions (UTRs) of their target genes to induce their cleavage or inhibit translation [10]. Previous researches were shown that miRNAs play crucial roles in diverse biological processes and diseases [11]. Some miRNAs have been confirmed to have bone remolding effects, miR-214-3p, miR-29b, and miR-34a [12–14]. In our previous study, sequencing the transcriptome in a rabbit model of bone infection found that miRNA-34a-5p played crucial role in the bone remodeling in infected bone nonunion animal models. In the previous studies, miR-34a had been proved to regulate tumor development, such as breast cancer, nonsmall cell lung cancer, colorectal cancer, and prostate cancer [15–18]. However, whether miRNA-34a-5p regulates bone formation after bone infection has not been explored.

We hypothesized that EHP promoted bone remolding in infected bone nonunion during post infection treatment, and its molecular mechanism was involved with miRNA-34a-5p. In this study, Micro-CT, and morphology detecting were carried out to reveal bone healing effect of EHP in infected bone nonunion animal models. We observed down-regulation of miRNA-34a-5p in LPS-induced osteoblasts, and verificated molecular mechanism by which EHP affected miRNA-34a-5p and regulated osteogenic factors. Thus, the aim of this study is to provide a new and supplementary remedy for bone remolding in infected bone nonunion during post infection treatment.

## Materials and methods

### Erxian herbal pair (EHP) extract preparation and chemical components identification

Two plant materials *Epimedium sagittatum* Maxim. and *Curculigo orchioides* Gaertn. were mixed with ratio of 1:1, according to clinical experience and TCM theory [19]. The dried materials were extracted with 10 x (v/w) distilled water at 100°C for 2 h twice. A freeze drier was used to lyophilize the water extracts. At last, the freeze-dry powder was kept at 4°C.

The UPLC-Q/TOF-MS (Ultra performance liquid chromatography-quadrupole-time of flight-mass spectrometer) system consisted of an AcquityTM ultra performance liquid chromatography (UPLC) system (Waters Corporation, Milford, MA, United States) and a Synapt G2 mass spectrometer (MS) (Waters MS-Technologies, Manchester, United Kingdom) equipped with an electro spray ion (ESI) source. Chromatography was performed according to the following parameter: Acquity UPLC BEH C_18_ column (2.1 × 150 mm, 1.7 µm, Waters Corporation, Milford, MA, United States), flow rate was 0.3 mL/min, column temperature was set as 40°C, mobile phases were set as A (HCOOH: CH_3_CN = 0.1: 100, v/v) and B (HCOOH: H_2_O = 0.1: 100, v/v): 0–14 min, 99–50% B; 14–15 min, 50–40% B; 15–15.5 min, 40–1% B; 15.5–18 min, maintained at 1% B.

For MS analysis, the ESI source was operated in both positive and negative ion modes with the following parameters: ion source gas 1 was 45 psi, ion source gas 2 was 55 psi, curtain gas was 35 psi, source temperature was 600°C, ion sapary voltage floating was 5500 V for positive ion mode and −4500 V for negative ion mode. The IDA MS/MS experiments acquired spectra in the high sensitivity mode with ± 60 psi declustering potential, 35 eV collision energy with ± 15 eV collision.

### Preparation of infected bone nonunion rabbit models

For preparation of infected bone nonunion rabbit models, we used New Zealand white male rabbits (SYXK 2019–0010), which were obtained from the Animal Experiment Center of Zhejiang Academy of Traditional Chinese Medicine. The rabbits (n = 10/group; age, 3 months, 3.0–3.5 kg weight) were housed in individual cages and offered the same feed and distilled water during the experimental period. The rabbit models were prepared according to established method [20,21]. Briefly, animals were punched a bone defect of 2 mm-diameter at tibia plateau. Subsequently, the holes were sealed with bone wax, and 1 × 10^6^ CFU/mL *S. aureus* suspension (China General Microbiological Culture Collection Center, CGMCC) was injected into the bone marrow by penetrating the bone wax layer. At the end of 4 weeks after infection, 90% rabbits were diagnosed with bone infection. Then animal groups and treatments timetable are indicated in Table 1. The rabbits in the Van-CS (vancomycin-calcium sulfate) and Van-CS+EHP (vancomycin-calcium sulfate+EHP) groups were debrided dead bone tissue, punched two adjacent 4-mm-diameter holes at tibia plateau, then implanted Van-CS in the holes. After 4 weeks of implanting Van-CS, rabbits in Van-CS+EHP group were administered EHP intragastrically at 384.56 mg/kg/day for 8 weeks. This administration dosage was confirmed by our preliminary experiments. All animal experiments were approved by the Animal Ethics Committee of Zhejiang Academy of Traditional Chinese Medicine (Approval No. [2019]004).

**Table 1.** Animal groups and treatment.

			Treatment(T)		
Group	Number	Model(M)	Vancomycin	EHP	Euthanasia time point after M or T
Control	5	None	None	None	4 w after M, 8 w after T
Model	5	Infection	None	None	4 w after M
	10	Infection	None	None	8 w after T
Van-CS	10	Infection	12.5 mg	None	8 w after T
Van-CS+EHP	10	Infection	12.5 mg	*384.56* mg/kg/day	8 w after T

### Evaluation of bone formation

At the end of the 8 weeks after treatment with EHP, blood samples were collected from the ear vein, white blood cell (WBC) counting was detected by Automated Hematology Analyzer (Sysmex, XN-1000 V, Japan), and alkaline phosphatase (ALP) level was detected by using ELISA kit (Beyotime Biotechnology Co. Ltd., Shanghai, China). The rabbits were euthanized and tibia specimens were examined by micro computed tomography (micro-CT, SkyScan-1172, Bruker, Switzerland). The indexes such as bone volume/tissue volume ratio (BV/TV) and bone mineral density (BMD) were analyzed to evaluate bone formation [22].

### Medicated serum harvesting and main ingredient quantification

In total, 40 SD rats (n = 20/group) were administered 2 mL of Erxian extract gastrogavage (150 mg/kg/d) once a day for 1 week to prepare Erxian-containing serum. The control animals were administered equal volumes of distilled water. Venous blood was taken from the abdominal aorta, after the last administration, and centrifuged at 4000 r/min for 15 min. Then the separated serum was inactivated in 56°C for 30 min.

For main ingredient quantification in EHP-containing serum (EHP-CS), 900 μL methanol-acetonitrile solution was added into 300 μL EHP-CS or blank serum (Con), then vortexed for 1 min, centrifuged at 14000 r/min for 15 min at 4°C to obtain supernatant. Standard substances of Baohuoside Ι and Orcinol Glucoside (Shanghai yuanye Bio-Technology Co., Ltd, Shanghai, China) were dissolved in methanol to make mother stock solutions. Further, combined spiking stock solutions of the two reference substances were prepared by stepwise dilution from the mother stock solutions, and filtered with 0.22 µm membranes. Then 2 µL volume solutions were injected into the UPLC-Q/TOF-MS system for analysis [23,24]. The UPLC conditions were the same as those previously described. For MS analysis, the ESI source was operated in negative ion modes,the parameters were set as followed: ion source gas 1 was 55 psi, ion source gas 2 was 55 psi, curtain gas was 35 psi, source temperature was 600°C, ion sapary voltage floating was −4500 V. MRM (multiple reaction monitoring) spectrum scanning method was used to acquire spectra in the high sensitivity mode, the MRM parameters for Baohuoside Ι and Orcinol Glucoside were listed in Table 2.

**Table 2.** Sequences of primers used in real-time polymerase chain reaction.

Gene	Forward primer	Reverse primer
Runx2	CATGGCCGGGAATGATGAG	TGTGAAGACCGTTATGGTCAAAGTG
ALP	CATCGCCTATCAGCTAATGCACA	ATGAGGTCCAGGCCATCCAG
BMP2	TGGAAGTGGCCCATTTAGAG	GCTTTTCTCGTTTGTGGAGC
SMAD2	CTCTCCGGCTGAACTGTCTC	GCCGTCTACAGTGAGTGAGG
β-actin	GGAGATTACTGCCCTGGCTCCTA	GACTCATCGTACTCCTGCTTGCTG
MiR-34a-5p	TGCGCTGGCAGTGTCTTAGCTG	CCAGTGCAGGGTCCGAGGTA
U6	CGCTTCACGAATTTGCGTGTCAT	CAAAGTGCTTACAGTGCAGGTAG

### Osteoblasts preparation and treatment

Osteoblasts were isolated from rat calvarias and cultured in incubator at 37°C, with 5% CO_2_ [25]. The blank serum group was given DMEM medium + blank serum; the model group was given DMEM medium + blank serum + LPS (10 μg/mL); the EHP-CS group was given DMEM medium + EHP containing serumat four different doses (2.5%, 5%, 10%, and 20%) + LPS (10 μg/mL). The results of MTT assays showed that when the EHP-CS was 10%, the drug had the best effect [26]. Subsequent experiments were conducted at this concentration.

In order to investigate effect of miRNA-34a-5p mimic/inhibitor and SMAD2 siRNA on osteoblasts, cells were first serum-deprived for 6–8 h, plated at 60–70% confluence and then transfected with SMAD2 siRNA (GenePharm Pharmaceutical Technology Co., Ltd., Shanghai, China) or miRNA-34a-5p mimic/inhibitor (Sangon Biotech Co., Ltd., Shanghai, China) for 6 h with Lipofectamine 2000 (Invitrogen, Waltham, MA, USA).

### Evaluation of cell proliferation

MTT assay was used to measure the proliferation rates of osteoblasts. Osteoblasts were dispensed into 96-well plate with 100 μL of fresh medium. At the end of the 24 h culture, a total of 50 μL of MTT solution was put into the cell and incubated at 37°C for4 h. Then, the solution was discarded, 100 μL DMSO was added to each well. At last, the absorbance was measured at 490 nm.

### ALP staining and alizarin red staining

ALP staining was performed following the ALP staining kit protocols (KGI Biotechnology Co., Ltd., JiangSu, China). For calcification induction, osteoblasts were cultured in induction medium for 14 days, and the medium was changed after 48 h. After induction, cells were fixed in 4% formaldehyde and washed with PBS, then stained with 0.1% alizarin red. The orange or red nodes were identified as calcium nodules [27].

### Dual-luciferase assay

HEK 293 cells were seeded into dark 96-well plates, and cultured in incubator at 37°C, with 5% CO_2_. Cells were transfected with 50 nM miRNA-34a-5p mimic or scrambled mimic and 400 ng of dual luciferase vector expressing the wild-type or mutant SMAD2 (GenePharm Pharmaceutical Technology Co., Ltd., Shanghai, China) by using the Lipofectamine 2000 (Invitrogen, Waltham, MA, USA). After 48 h of incubation, luciferase reporter gene assay was performed using a dual-luciferase reporter assay kit (Yeasen Biotechnology Co., Ltd., Shanghai, China) [27]. The ratios of renilla luciferase activity to firefly luciferase activity were calculated to evaluate target relationship of miRNA-34a-5p and SMAD2.

### qRT-PCR analysis

Total RNA of osteoblasts was extracted with 0.5 mL TRIzol reagent (Invitrogen Life Technologies, Carlsbad, CA, United States). The quality of the extracted RNA was identified with a spectrophotometer (NanoDrop 2000, Thermo Scientific, MA, United States), and RNA purity was determined by the A260/280 ratio range of 1.8–2.1. All RNA samples were stored at −80°C for the further expriments. Reverse transcription was carried out by using a cDNA first strand synthesis kit (Bioer Technology Co., Ltd., Hangzhou, China), and quantitative reverse transcription PCR were performed by using SYBR Green I real time PCR kit (Bioer Technology Co., Ltd., Hangzhou, China). MiRNAs were validated with reverse transcription kit and qRT-PCR quantitation kit (Sangon Biotech Co., Ltd., Shanghai, China). The fluorescence quantitative PCR reaction system was performed as followed: (1) mRNA: 10 μL SYBR Premix (Diamond, USA), 1 μL each the upstream and downstream primers, 2 μL cDNA and 6 μL ddH_2_O; (2) miRNA: 10 μL 2*miRNA qPCR master mix, 0.5 μL forward Primer (10 μM), 0.5 μL reverse Primer (10 μM), 1 μL ROX Reference (L), forward Primer (10 μM), 2 μL cDNA and 6 μL ddH_2_O. The reaction was carried out on a 7500 fluorescent quantitative PCR instrument (Applied biosystems, USA). The amplification program was as follows: (1) mRNA: 94°C for 2 min, 94°C for 10s, 60°C for 15s, 72°C for 30s, 40 cycles; (2) miRNA: 95°C for 30s, 95°C for 5s, 60°C for 30s, 72°C for 30s, 40 cycles. MiRNA expression was normalized to U6 expression, and the fold changes of mRNAs and miRNAs in each group were normalized to the control groups. Primers sequences in this study were listed in Table 2. The expression of detected genes was analyzed using the 2^−ΔΔ^ CT method.

### Western blot analysis

In brief, osteoblasts were lysed in RIPA lysis buffer (KeyGEN BioTech Co., Ltd., Jiangsu, China), and total proteins in osteoblasts were quantified using BCA Protein Assay kit (KeyGEN BioTech Co., Ltd., Jiangsu, China). The proteins were subjected to 12% or 10% SDS-PAGE and were then transferred to 0.45-µm PVDF membranes (Bio-Rad Co., Ltd., CA, USA), which were blocked with 5% BSA in PBST for 1 h. After incubation with specific primary antibodies at 4°C overnight, HRP-conjugated secondary antibodies were added. Then the PVDF membranes were visualized in ChemiDocTM MP Imaging system (BioRad Co., Ltd., CA, USA) using a chemiluminescent ECL reagent (BioRad Co., Ltd., CA, USA). Anti-BMP2 polyclonal rabbit antibody, anti-ALP polyclonal rabbit antibody (bs-522526, 1:1000, BiossBiotechnology Co.,Ltd.,Beijing, China), and anti-SMAD2 polyclonal rabbit antibody (bs-0718 R, 1:500, Bioss Biotechnology Co., Ltd., Beijing, China) served as primary antibodies. GAPDH (ab181602, 1:1000, Abcam, Cambridge, USA,) was used as control.

### Statistical analysis

The data are presented as the mean ± SD, SPSS software 22.0 (SPSS, Inc., Chicago, IL, United States) was used for statistical analyses. Student’s t-test was used to analyze the comparison between two groups of unpaired data with normal distribution and homogeneity of variance. One-way analysis of variance (ANOVA) and Tukey’s post-hoc test were used to compare between multiple groups, *p < *0.05 was considered as significant.

## Results

Here, we hypothesized that EHP promoted bone repair after antibiotic treatment, by increasing bone volumn and bonemineral density. MiRNA-34a-5p promoted the proliferation and mineralization abilities of osteoblasts, by inhibiting target gene SMAD2. Moreover, EHP reversed the LPS inhibition in osteoblasts, by increasing miRNA-34a-5p expression. The results suggested that targeting the miRNA-34a-5p/SMAD2 axis might be a new therapeutic strategy in treatment of bone nonunion after anti-infection treatment in infected bone nonunion.

### Chemical constituents characterization in Erxian herbal pair (EHP) extract

**Figure 1.**
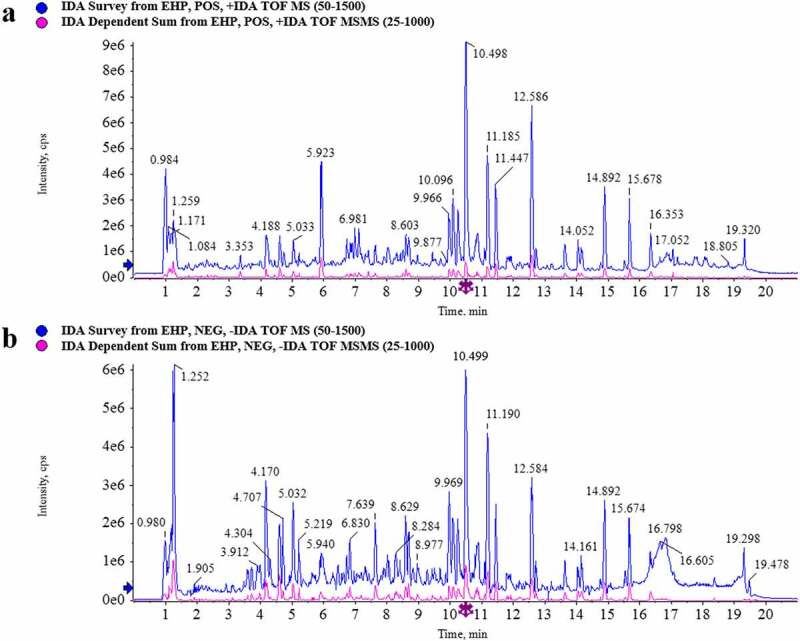
TIC of EHP extract of 0–20 min. (a) Postive mode. (b) Negative mode.

The composition profiles of EHP extract were analyzed by UPLC-Q/TOF-MS. The element compositions were calculated and confirmed by using MarkerLynx (4.1) software. Total ion chromatogram (TIC) was obtained in the positive ion and negative ion modes (Figure 1). In comparison with the database of TCM MS/MS Library of SCIEX OS software, 38 constituents were assigned in the positive ion mode (Supplementary table S1), and 53 constituents were assigned in the negative ion mode (Supplementary table S2). A total of 79 constituents in EHP extract were identified by combining the constituents of positive and negative ion mode and removing duplicate ones (Supplementary table S3). As the results shown, Baohuoside I, Curculigoside, Epimedin A, Epimedin B, Epimedin C, Icarrin, Orcinol glucosid, Quinic acid were the main constituents in EHP extract.

### Erxian herbal pair (EHP) promoted bone formation

**Figure 2.**
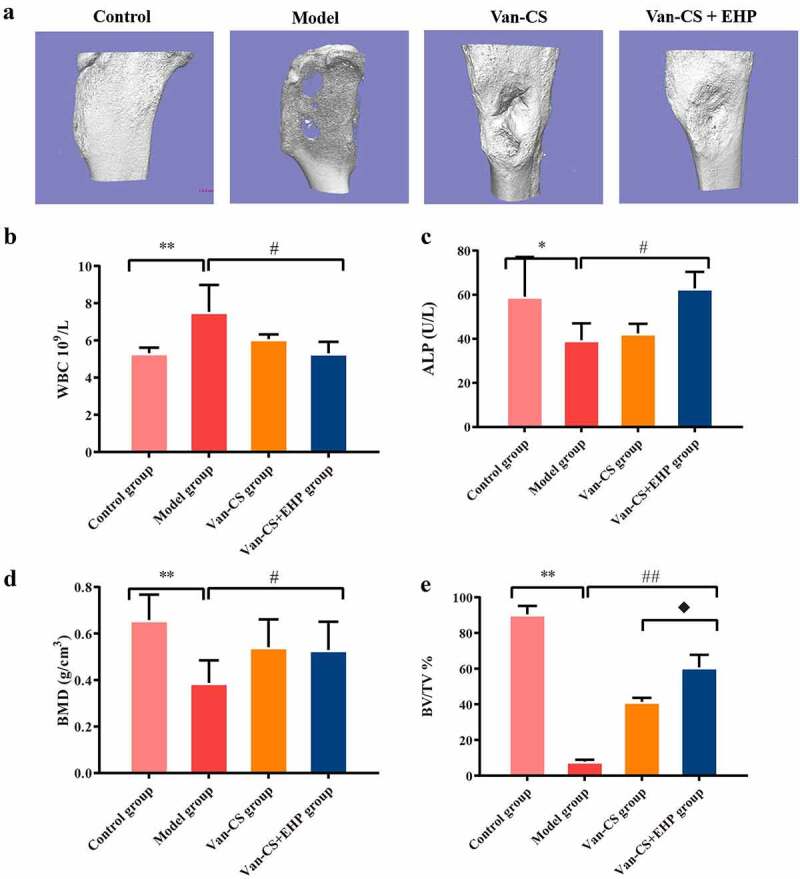
EHP promoted bone formation in the rabbit after infection with *Staphylococcus aureus*. (a)Three-dimensional reconstruction of bone defects of the rabbit tibia 8 weeks after infection with *Staphylococcus aureus* and treatment with Van-CS+EHP. (b and c) The results of WBC and ALP activity assays in the rabbit serum were performed at the eighth week after treatment. (d and e) Microarchitecture of the rabbit tibias 8 weeks after treatment with Van-CS+EHP. Data depict the mean ± standard deviation (mean ± SD) and are representative of three independent experiments. *P< 0.05, **P< 0.01 compared with the control groups; ^#^*P< *0.05, ^##^*P < *0.01 compared with the model groups; ♦*P< *0.05 compared with the Van-CS groups.

It can be seen that some rabbits gradually weakened, with a decreased appetite, after modeling process. Most rabbits had obvious tissue swelling around the calf wound, with purulent secretions and white and yellowpus overflowing from the wounds, and there was no hair growth around the wound, thus confirming that infected bone models had been established. At the end of the 8 weeks after treatment with EHP, the rabbits in model group had severe bone infection. The tibia plateaus of the Van-CS+EHP groups seemed flatter than those of the Van-CS groups (Figure 2(a)). The WBC levels in the model group were higher than control group (*P*< 0.01), WBC levels in Van-CS and Van-CS+EHP treatments decreased than the model group, and Van-CS+EHP treatments significantly inhibited WBC levels (*P*< 0.05, Figure 2(b)). ALP in serum decreased in the model groups, compared with control groups (*P*< 0.05), and was improved by Van-CS+EHP at the end of the 8 weeks after treatment, compared with model groups (*P*< 0.05,Figure 2(c)). With 8 weeks treatment, BV/TV and BMD in control groups were significantly higher than the model groups (*P*< 0.01), which indicated *s.aureus* infection caused the number of bone decreasing. Moreover, BV/TV indicator in Van-CS+EHP group was higher than that in the Van-CS group significantly. These results indicated EHP could noticeably increase bone volumns (Figure 2(d,e)).

### Quantification of ingredients in Erxian herbal pair-containing serum

**Figure 3.**
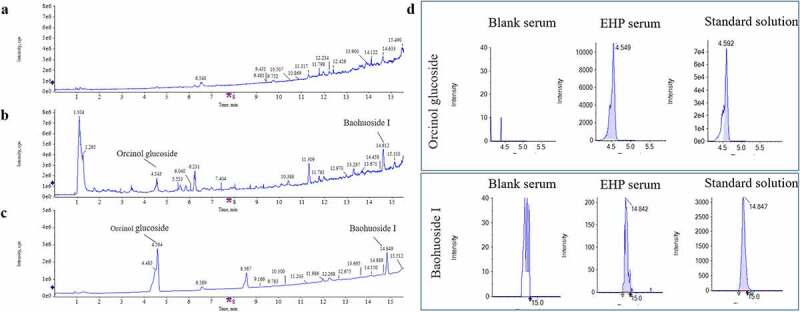
Typical chromatograms of Baohuoside Ι and Orcinol Glucoside in EHP-CS. (a) MRM of Blank serum. (b) MRM of EHP-CS. (c) MRM of standard. (d) Chromatograms of Baohuoside Ι and Orcinol Glucoside in blank serum, EHP-CS and standard solution.

As the UPLC-Q/TOF-MS results of EHP-CS shown, Baohuoside Ι and Orcinol Glucoside were main ingredients that absorbed in blood. The established quantitative method was applied to determine the contents of main ingredients Baohuoside Ι and Orcinol Glucoside in EHP-CS. The ingredients quantification results were concluded by matching the accurate mass data of references substances. The Linear parameters and concentrations of the two ingredients in serum were listed in Table 3 and Table 4. Typical chromatograms of the two ingredients were shown in Figure 3. In our following study, Baohuoside Ι and Orcinol Glucoside were considerated as quality indexes for EHP-CS.

**Table 3.** Linearity and concentration for the two ingredients.

Compound name	Formula	Standard curve	Linear range (ng/mL)	r	Area	Actual Concentration (ng/mL)
Baohuoside Ι	C_27_H_30_O_10_	y = 722521x – 1726.4	3.2–400	0.9999	6.28E+02	3.258
Orcinol Glucoside	C_13_H_18_O_7_	y = 742612x – 3107.2	3.2–400	0.9999	4.61E+04	66.222

**Table 4.** The MRM parameters.

Compound	Adduct	Precursor ion(m/z)	Fragment (m/z)	DP (V)	CE (V)	Accumulation (sec)	Retention time(min)
Orcinol Glucoside	M+ HCOOH-H	331.10	123.0453	−80	−25	0.1	4.6
Baohuoside Ι	M-H	513.18	366.1107	−80	−35	0.1	14.9

### Erxian herbal pair (EHP) reversed the inhibition of osteoblasts caused by LPS

**Figure 4.**
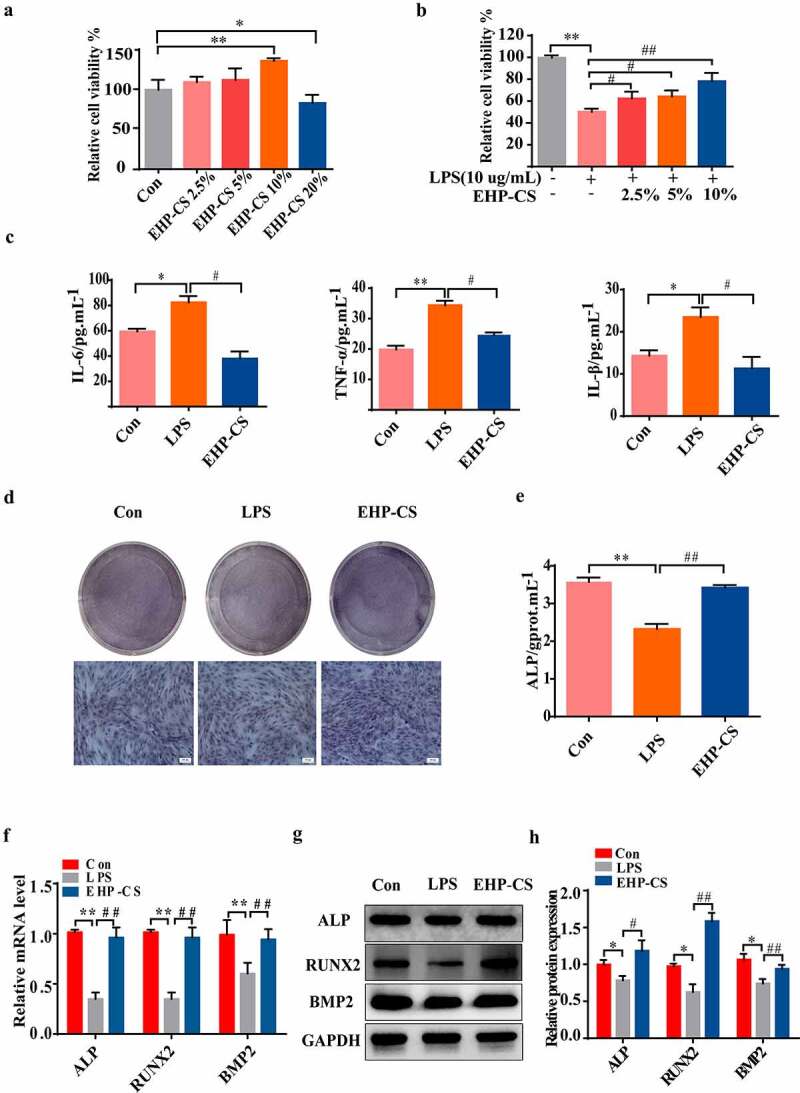
LPS inhibited osteoblast proliferation and differentiation, and EHP-CS controlled the effect of LPS on osteoblast differentiation. (a and b) Rat osteoblasts were treated with or without LPS and different concentrations of EHP-CS, and the survival rate of osteoblasts was measured by MTT. (c) IL-6, IL-1β, and TNF-α activity levels were detected by ELISA. (d and e) ALP staining and activity assays were performed on day 2. (f) RT-PCR was performed to analyze the expression levels of RUNX2, ALP,and BMP2 mRNA. (g and h) western blot analysis showed the protein levels of RUNX2, ALP, and BMP2. Data depict the mean ± standard deviation(mean ± SD) and are representative of three independent experiments. *P< 0.05, **P< 0.01 compared with the control groups; ^#^*P < *0.05, ^##^*P < *0.01 compared with the model groups.

The survival rate of osteoblast cells after intervention with EHP-CS showed that doses of 2.5%, 5% and 10% EHP-CS had no cytotoxicity to osteoblasts, and 20% EHP-CS had inhibitory effect to osteoblasts (*P*< 0.05, Figure 4(a)). Therefore, EHP-CS doses of 2.5%, 5% and 10% were selected for further experiments. The survival rates of osteoblasts in the model group were decreased significantly. In osteoblasts treated with EHP-CS, the cell morphology and cell proliferation exhibited considerable changes, and the 10% EHP-CS group had the best effect (*P*< 0.01, Figure 4 (b)). Therefore, subsequent experiments were conducted at this concentration. LPS at 10 μg/mL significantly increased the IL-6, IL-β, and TNF-α expression levels in osteoblasts. EHP-CS was reduced to approximately 80% compared with the model group (*P*< 0.05, Figure 4(c)). As shown in Figure 4 (d) and (e), (*P*< 0.01), 10% EHP-CS distinctly increased ALP activity and staining, and the ALP activity was almost 1.3 times that of the model group. The qRT-PCR data show that the mRNA expressions of ALP, RUNX2, and BMP2 were decreased in LPS groups. Osteoblasts co-cultured with LPS and EHP-CS significantly enhanced the mRNA levels of all these four genes (*P*< 0.01, figure 4(f)). The Western Blotting results shown that the inhibition protein expressions of BMP2, ALP, and Runx2 induced by LPS were rescued by EHP-CS (*P*< 0.01, *P*< 0.05, *P*< 0.01, Figure 4(g,h)). Taken together, EHP treatment can counteract the inhibitory effect of LPS on osteoblasts.

### Erxian herbal pair (EHP) regulated osteoblasts by increasing miRNA-34a-5p expression

**Figure 5.**
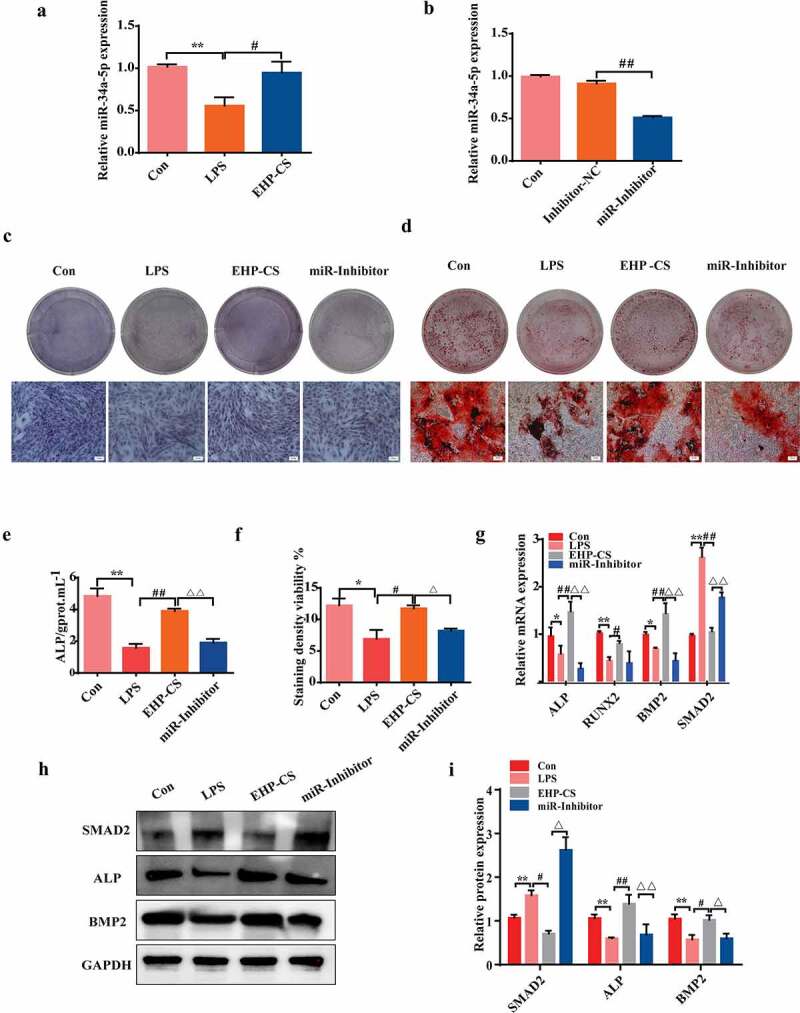
Effects of EHP-CS on RUNX2, ALP, and SAMD2 mRNA and protein expression in LPS-induced osteoblasts after inhibition of miRNA-34a-5p. (a) Relative level of miRNA-34a-5p in rat osteoblasts treated with 10 μg/mL LPS and LPS with 10% EHP-CS. (b) Stem-loop RT-PCR was performed to analyze the expression of miRNA-34a-5p after transfection with a miRNA-34a-5p-specific inhibitor. (**C** and**E**) ALP staining and activity assays were performed on day 2 of drug intervention. (d and f) Alizarin red S staining and activity assays were performed on day 14 of drug intervention. (g, h and i) RT-PCR and western blotting were performed to analyze the mRNA and protein levels of osteogenic-specific markers after miRNA-34a-5p inhibitor transfection. Data depict the mean ± standard deviation (mean ± SD) and are representative of three independent experiments. *P< 0.05, **P< 0.01 compared with control groups; ^#^*P*< 0.05, ^##^*P*< 0.01 compared with model groups; ^Δ^*P*<0.05, ^ΔΔ^*P*<0.01 compared with EHP-containing groups.

We measured miRNA-34a-5p expression in LPS-induced inflammatory responses of osteoblasts by using qRT-PCR. As shown in Figure 5(a), miRNA-34a-5p was dramatically down regulated after LPS application, however, EHP-CS up regulated miRNA-34a-5p levels (*P*< 0.05). To investigate biological function of miRNA-34a-5p in osteogenic differentiation, osteoblasts were transfected with miRNA-34a-5p inhibitor for 6 h, and then co-cultured in the presence of 10 μg/mL LPS and 10% EHP-CS. QRT-PCR results confirmed that miRNA-34a-5p inhibitor effectively decreased miRNA-34a-5p expressions in osteoblasts, compared with inhibitor N.C group (*P*< 0.01, Figure 5(b)). As well, ALP expressions and activities were decreased by the miRNA-34a-5p inhibitor (*P*< 0.01, Figure 5(c,e)). Alizarin red staining results indicated that osteoblasts mineralization abilities were decreased under miRNA-34a-5p inhibitor treatment (*P*< 0.05, Figure 5(d,f)). QRT-PCR analysis showed that miRNA-34a-5p inhibitor-infected osteoblasts exhibited lower levels of ALP, RUNX2 and BMP2 than that in the EHP group (Figure 5(g)). Western blot analysis indicated thatmiRNA-34a-5p inhibitor transfecting dramatically downregulated the protein levels of ALP and BMP2 (*P*< 0.01, *P*< 0.05, Figure 5(h,i)).

### MiRNA-34a-5p directly targets SMAD2

**Figure 6.**
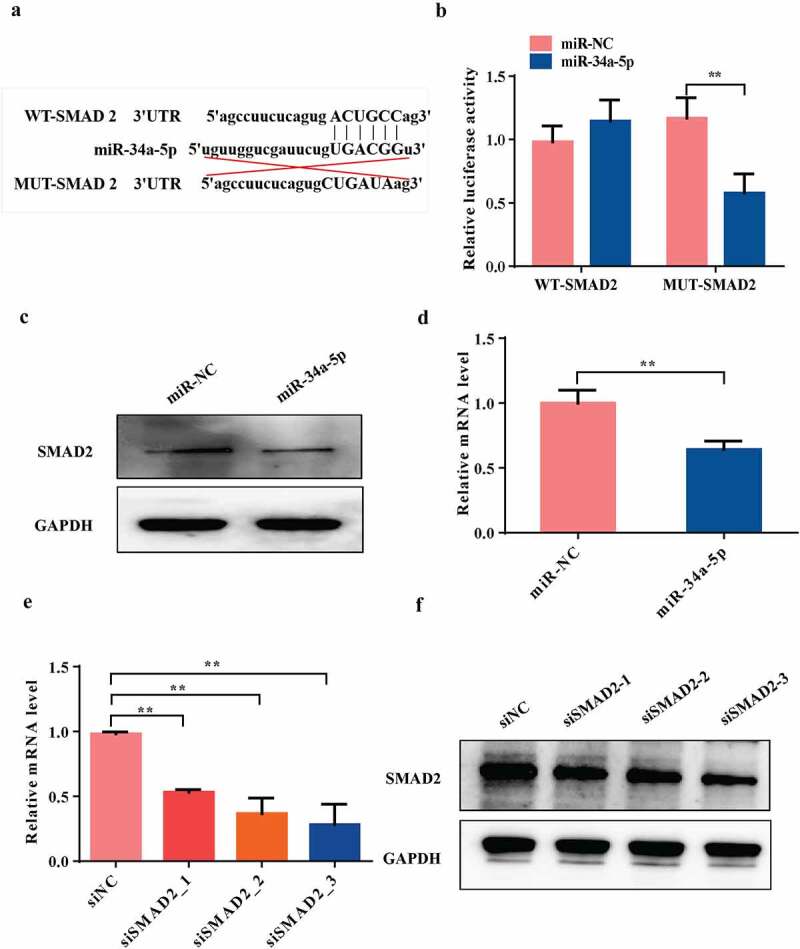
SMAD2 is a direct target of miRNA-34a-5p.(a and b) Diagram of putative miRNA-34a-5p binding sequence in SMAD2 3'UTR and its mutant in luciferase reporter assay. A luciferase reporter assay was performed to measure luciferase activity in osteoblast cells; WT = wild-type, MUT = mutant-type. (c and d) Western blot and qRT-PCR analyses of the expression of SMAD2 after overexpression of miRNA-34a-5p. (e and f) The knockdown efficiency of three SMAD2 siRNAs was confirmed by qRT-PCR and western blot. Data depict the mean ± standard deviation (mean ± SD) and are representative of three independent experiments. *P< 0.05; **P< 0.01 compared with the control groups.

We used TargetScan (http://www.targetscan.org/vert_80/) to predict the targets genes of miRNA-34a-5p. Among the candidate genes, osteoblast differentiation-related gene SMAD2 has miRNA-34a-5p binding sites in its 3'UTR (Figure 6(a)). The Dual luciferase reporter analysis showed that overexpression of miRNA-34a-5p significantly inhibited the luciferase reporter activity of the vector containing the WT SMAD2 3'UTR (*P*< 0.01, Figure 6(b)). The qRT-PCR and western blot results indicated that the mRNA and protein expression levels of SMAD2 in osteoblast cells were significantly decreased by transfecting with overexpression of miRNA-34a-5p (*P*< 0.01, Figure 6(c,d)). In order to confirm SMAD2 was a direct target gene of miRNA-34a-5p, we suppressed the expression of SMAD2 by transfecting osteoblasts with siRNAs against SMAD2. The data showed that the mRNA and protein levels of SMAD2 was significantly suppressed by transfected with siRNA SMAD2-3 (5'GCCUAAGUGAUAGUGCGAUTT3’) (*P*< 0.01, Figure 6(e,f)). Therefore, siRNA SMAD2-3 was chosen for the subsequent functional experiments. Taken together, our data suggested that SMAD2 could be downstream target gene of miRNA-34a-5p in osteoblast cells.

### siRNA SMAD2 remedy reduction effect of miRNA-34a-5p on osteogenesis

**Figure 7.**
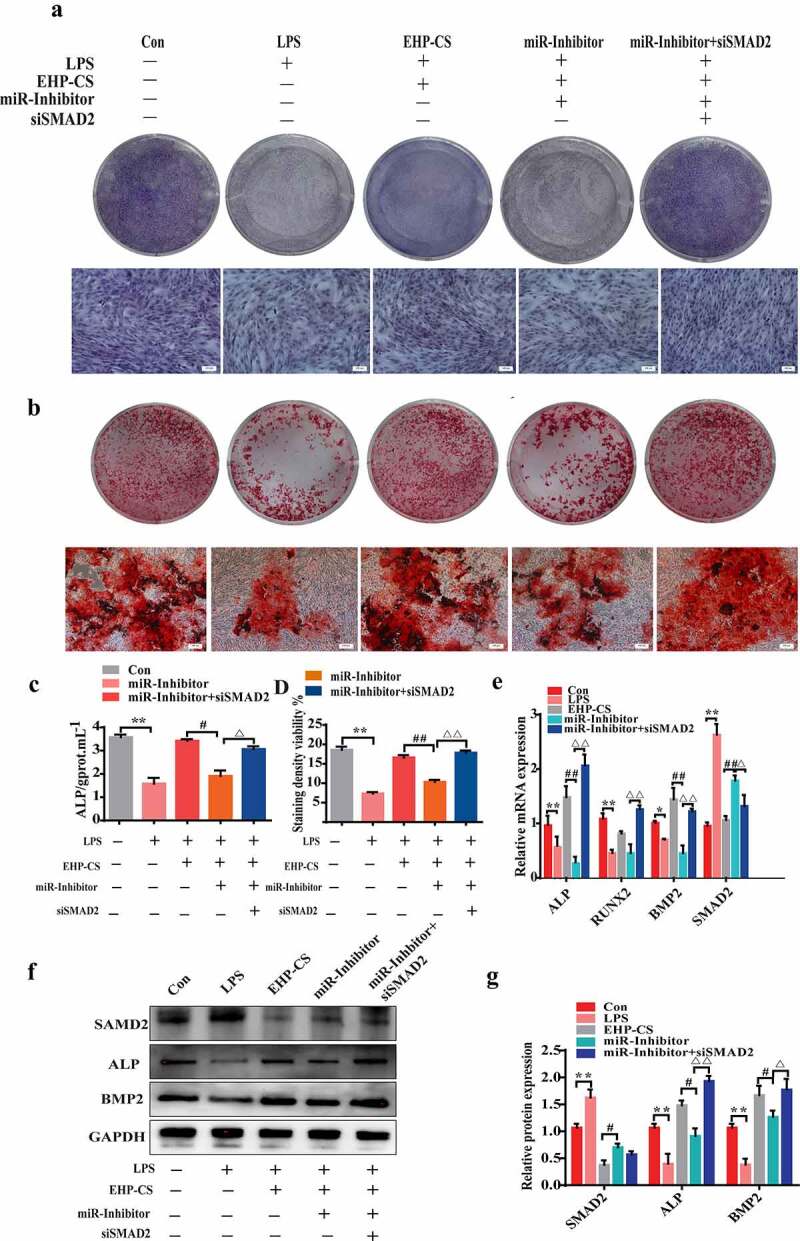
SMAD2 knockdown reverses the effect of the miRNA-34a-5p inhibitor on osteogenesis in LPS-induced bone loss. (**A** and**C**) ALP staining and activity assays were performed on day 2 of drug intervention. (b and d) Alizarin red S staining and activity assays were performed on day 14 of drug intervention. (**E, F** and **G**) western blotting and qRT-PCR were used to analyze osteogenic factor protein and mRNA expression after different treatments. Data depict the mean ± standard deviation (mean ± SD) and are representative of three independent experiments. *P< 0.05; **P< 0.01 compared with the control group; ^#^*P*< 0.05, ^##^*P*< 0.01 compared with the EHP-containing groups; ^Δ^*P*<0.05, ^ΔΔ^*P*<0.01 compared with the miR-inhibitor groups. LPS groups were treated with LPS, EPH-CS groups were treated with EPH-CS, miR-Inhibitor groups were transfected with miRNA-34a-5p inhibitor for 6 h, and then treatedwith LPS and EPH-CS, miR-Inhibitor+siSMAD2 groups were transfected with miRNA-34a-5p inhibitor and siSMAD2 for 6 h, and then treatedwith LPS and EPH-CS.

We further confirmed that the effect of miRNA-34a-5p during the LPS-induced bone loss mechanism was mediated by targeting SMAD2. Osteoblasts were silenced miRNA-34a-5p with miRNA-34a-5p inhibitor and knocked down SMAD2 with siRNA SNAD2 for 6 h, and then treated with 10 µg/mL LPS and EHP-CS at 10%. Compared with miR-inhibitor, after SMAD2 knockdown, ALP staining and activity were significantly up regulated (*P*< 0.05, Figure 7(a,c)). As well, siRNA SMAD2 was proved to reverse the inhibition effect of miRNA-34a-5p inhibitor on osteogenesis differentiation, as examined by alizarin red staining, and markedly increased mineralized nodule formation at 14 days (*P*< 0.01, Figure 7(b,d)). The mRNA and protein expression levels of ALP and BMP2 were dramatically up regulated in SMAD2 knockdown cells (*P*< 0.01, Figure 7(e–g)). Further, EHP counteracted LPS-induced osteoblasts suppression by increasing miRNA-34a-5p expression, which regulates osteogenesis by directly targeting SMAD2.

## Discussion

*Staphylococcus aureus* (*S. aureus*) is the most common bacterial species involved in infected bone nonunion. The inflammatory response caused by bacteria induces imbalance between bone resorption and bone formation, which can lead to bone nonunion and fracture healing delay [28]. The bone formation process is the process of balancing the activity of osteoblasts and osteoclasts. Chronic persistent inflammation can stimulate bone resorption. The increased expression of TNF-α has been proved to promote osteoclastic differentiation from bone marrow mesenchymal stem cells and bone resorption [29]. IL-1 inhibits the production and function of osteoblasts [30]. At present, there is still a lack of prevention or intervention methods for bone loss.

*Curculigo orchioides* Gaertn. is a well-known Chinese herbal medicine and is considered a major active compound [31]. It has been used to promote bone healing, which preventsosteoporosis by stimulating osteoblasts proliferation and differentiation, as well as attenuating adipogenic differentiationfrom mesenchymal stem cells [32]. Epimedin A, epimedin B, epimedin C and icariin are major bioactive compounds in *Epimedium sagittatum* Maxim. *Epimedium sagittatum* Maxim. can significantly increase the expression of apoptotic proteins of osteoclasts and protective proteins of osteoblasts and enhance the BMD of femoral heads, preventing osteoporosis and leading to collapse [33]. In previous studies, Erxian formula consisting of *Curculigo orchioides* Gaertn. and *Epimedium sagittatum* Maxim. was shown to be the major contributor to preventing osteoblast apoptosis [7], promoting osteoblast proliferation [34], and self-renewal and osteoblastic differentiation of bMSCs [35].

As the ingredients profile of EHP-CS shows, the main ingredients Baohuoside Ι and Orcinol Glucoside were detected in the EHP-CS with high bioavailability. Although the polarity of Orcinol Glucoside is very high, the molecular weight of Orcinol Glucoside is small, so it can enter the blood through the cell membrane. Baohuoside Ι is the main metabolite of Icariin, Epimedin A, Epimedin B and Epimedin C, and is also one of the main components absorbed into the blood [36]. Orcinol Glucoside was shown to promote bone repair by increasing osteoblasts differentiation genes and decreasing adipogenic differentiation related genes from BMSCs. Baohuoside Ι was shown to be related to the induction of BMSCs differentiation into osteoblasts [37]. It was speculated that Baohuoside Ι and Orcinol Glucoside may be effective components in the EHP-CS. Therefore, we chose these two ingredients as quantitative indicators of the EHP-CS to ensure the homogeneity of EHP-CS.

The present study demonstrated that EHP have effect on bone repair in *S. aureus*-induced rabbit infected bone nonunion model. *In vivo*, micro-CT results suggested bone destruction and increased sequestration in the model group and increased infected bone nonunion. Van-CS+EHP provide a good environment for the elimination of inflammation and bone formation. EHP treatment increased BMD in the area of the bone defect and restored bone tissue by promoting volumes of new bone around the bone defect areas when compared with Van-CS groups at 8 weeks. ALP activity is a phenotypic marker of mature osteoblasts [38]. Therefore, we chose ALP as an index to evaluate changes in osteogenic capacity. The decreasing serum ALP levels in the model group indicated the inhibitory effects of bacterium on metabolic activityof osteoblasts. This shows that *S.aureus* infection inhibits the osteogenesis reaction, resulting in bone mass. However, EHP treatment increased ALP levels after antibiotic administration and accelerated bone union. However, the specific mechanism is unclear, and we are evaluating EHP-mediated bone remodeling at the cellular level.

MiRNAs are powerful modulators of fracture healing in the previous researches. MiRNA-223-3p is highly expressed in fracture patients, and regulates osteoblast cell by targeting fibroblast growth factor receptor 2 [39]. MiR-874-3p has been proved to promote the proliferation and differentiation of hBMSCs by downregulating the expression of target gene, thus may be used as a novel strategy for treatment of osteoporosis [40]. In our study, we speculated that miRNA-34a, a crucial regulator of osteoclast bone resorption and stem cell osteogenic differentiation, played an important role in new bone growth and bone repair. As the recent literatures shown, miRNA-34a inhibits osteoclasts differentiation by inhibiting the OPG/RANK/RANKL pathway. Additionally, miR-34a was previously found to improve osteoblastic differentiation and to promote new bone volumes [41]. MiRNA-34a can promote the osteogenic differentiation of BMSCs and increase the expression of the osteogenic differentiation marker genes RUNX2 and OCN, ALP activity and matrix mineralization ability. In addition, inhibition targeting of miRNA-34a in hMSCs increased bone formation in heterotopic bone formation mice models [42].

In our study, we carried out LPS-induced osteoblasts. LPS, a component of the outer membranes of gram-negative bacteria, has been verified to be capable of inducing bone resorption and inhibiting osteoblasts differentiation and function *in vivo* or *in vitro* [43]. LPS stimulates osteoblasts to secrete inflammatory cytokines, such as interleukin-6 (IL-6), prostagrandin (PG) E2 and receptoractivator of nuclear factor-kappa B ligand (RANKL), all of which induce osteoclasts activation [44], Furtherore, several recent studies revealed that the expression of IL-6, tumor necrosis factor-α (TNF-α) were increased by LPS stimulation in osteoblast, and osteogenic differentiation of osteoblasts is inhibited by LPS, which may be consideredas mechanism of inflammation induced bone loss [45,46]. To sum up, LPS-induced osteoblasts injury model was constructed in this study to investigate the mechanism of EHP on osteoblasts. As the results shown, miRNA-34a-5p was dramatically down regulated after treatment with LPS, and EHP restored the expression level of miRNA-34a-5p to normal. The improvement of osteogenic differentiation by EHP treatment was inverted by transfecting with miRNA-34a-5p inhibitor. The expressions of ALP and BMP2 were significantly reduced, and ALP activity analysis. Also, alizarin red staining results exhibited a trend consistent with the qPCR results that EHP treatment significantly reversed mineral nodule formation by down regulating miRNA-34a-5p in osteoblasts. It was concluded that EHP counteracted LPS-induced bone loss by up-regulating miRNA-34a-5p expression.

It is well known that miRNAs play a role in regulating the target genes. TargetScan prediction implied that SMAD2 was target gene of miRNA-34a-5p. Tranasforming growth factor β (TGF-β) signaling and BMP superfamily pathways have been proved to regulate osteoblasts proliferation and differentiation. SMAD2 is a member of the SMAD protein family, which play important role in BMP pathways [47]. Activated receptor-regulated SMADs (R-SMADs) include SMADs 1, 2, 3, 5 and 8. Both SMAD2 and SMAD3 are essential for chondrogenesis *in vivo*, SMAD3 suppresses chondrocyte hypertrophy, and overexpression of SMAD2 can regulates chondrocyte maturation [48]. In addition, activation of SMAD2/3 and p38 MAPK can increase the transcriptional activity of Runx2 and then enhance the osteoblastic differentiation of mesenchymal stem cells [49]. During osteogenesis, increased p-SMAD2 and decreased p-SMAD1 were observed in MSCs [50]. The previous studies indicated that the relationships between SMAD protein osteoblasts proliferation and bone healing.

In the present study, we concluded that miRNA-34a-5p directly regulated SMAD2 in osteoblast differentiation, and down regulation of SMAD2 up regulated BMP2 expression, while knockdown of SMAD2 rescued the inhibitory effect of miRNA-34a-5p on osteogenesis. EHP counteracted LPS-induced bone loss by suppressing SMAD2 expression. However, we did not clarify whether miRNA-34a-5p have effect on the other genes in TGF-β signaling pathway in this study, which should be verified in subsequent experiments. Whether EHP directly regulates the BMP2/Runx2 pathway by suppressing the expression of SMAD2 regulates bone formation needs to be further investigated.

## Conclusions

In summary, our data indicate that EHP can increase the number of osteoblasts to promote bone formation ininfected bone nonunion rabbit models after anti-infection treatment. MiRNA-34a-5p has a positive function in osteogenesis, and EHP promotes bone remodeling and osteoblast differentiation by increasing miRNA-34a-5p levels and osteogenic gene expression. By targeting SMAD2, miRNA-34a-5p rescued LPS-induced BMP2 down regulation. All the results implied that EHP has potential as a therapeutic agent in the treatment of bone nonunion after anti-infection treatment in infected bone nonunion.

## Supplementary Material

Supplemental Material

## Data Availability

The datasets used and/or analyzed during the current study are available from the corresponding author on reasonable request. For original data, please contact shoudanok@163.com.

## Abbreviations

BV/TV: Bone volume/tissue volume ratio

BMD: bone mineral density; EHP: Erxian herbal pair

EHP-CS:EHP-containing serum

LPS: lipopolysaccharide; S. aureus: Staphylococcus aureus

TIC: Total ion chromatogram

UPLC-Q/TOF-MS: Ultra performance liquid chromatography-quadrupole-time of flight-mass spectrometer

Van-CS: vancomycin-calcium sulfate.
