# Supplementary material for: Erxian herbal pair enhances bone formation in infected bone nonunion models and attenuates lipopolysaccharide-induced osteoblastinhibition by regulating miRNA-34a-5p
Source: Bioengineered. 2023 Jan 24;13(6):14339–56. doi: 10.1080/21655979.2022.2085388 (PMC9995130; doi:10.1080/21655979.2022.2085388)
Supplement: Supplemental Material [file KBIE_A_2085388_SM9510.zip › supplementary/supplementary table 1.docx]

Table S1 Characterization of chemical constituents of EPH by UPLC-Q/TOF-MS (Positive Mode).

| NO. | Component Name | Adduct | Area | Retention Time | Formula | Precursor Mass | Found At Mass | Mass Error (ppm) | Library Score | Isotope Ratio Difference |
| --- | --- | --- | --- | --- | --- | --- | --- | --- | --- | --- |
| 1 | L(+)-Arginine | M+H | 1501000 | 1.1 | C_6_H_14_N_4_O_2_ | 175.119 | 175.1187 | -1.4 | 95.8 | 3.5 |
| 2 | Alanine | M+H | 31590 | 1.11 | C_3_H_7_NO_2_ | 90.055 | 90.055 | 1 | 97.7 | 1.7 |
| 3 | L-Carnitine | M+H | 306700 | 1.14 | C_7_H_15_NO_3_ | 162.112 | 162.1124 | -0.5 | 96.2 | 2.4 |
| 4 | Glutamic acid | M+H | 77320 | 1.14 | C_5_H_9_NO_4_ | 148.06 | 148.0604 | 0 | 96 | 1.9 |
| 5 | Betaine | M+H | 392900 | 1.16 | C_5_H_11_NO_2_ | 118.086 | 118.0862 | -0.4 | 100 | 1.7 |
| 6 | Trigonelline | M+H | 425800 | 1.2 | C_7_H_7_NO_2_ | 138.055 | 138.0548 | -1.2 | 96.8 | 3 |
| 7 | Proline | M+H | 328600 | 1.23 | C_5_H_9_NO_2_ | 116.071 | 116.0706 | 0.2 | 98.5 | 0.5 |
| 8 | Cytidine | M+H | 45340 | 1.28 | C_9_H_13_N_3_O_5_ | 244.093 | 244.0926 | -1 | 100 | 6.5 |
| 9 | Nicotinic acid | M+H | 56580 | 1.75 | C_6_H_5_NO_2_ | 124.039 | 124.0393 | -0.1 | 97.2 | 1.8 |
| 10 | Nicotinamide | M+H | 91930 | 1.88 | C_6_H_6_N_2_O | 123.055 | 123.0553 | -0.2 | 99.6 | 0.7 |
| 11 | Adenosine | M+H | 173200 | 2.48 | C_10_H_13_N_5_O_4_ | 268.104 | 268.1037 | -1.1 | 100 | 5.1 |
| 12 | Guanosine | M+H | 101900 | 2.61 | C_10_H_13_N_5_O_5_ | 284.099 | 284.0989 | -0.2 | 99.1 | 4.1 |
| 13 | Phenylalanine | M+H | 420400 | 3.35 | C_9_H_11_NO_2_ | 166.086 | 166.0861 | -0.6 | 99.8 | 1.7 |
| 14 | Salidroside +NH_3_ | M+H | 61600 | 3.8 | C_14_H_20_O_7_.NH_3_ | 318.155 | 318.1545 | -0.8 | 95.9 | 6.6 |
| 15 | Higenamine | M+H | 29330 | 4.44 | C_16_H_17_NO_3_ | 272.128 | 272.1282 | 0.1 | 86.8 | 0.9 |
| 16 | Esculin hydrate | M+H | 5183 | 4.52 | C_15_H_16_O_9_ | 341.087 | 341.0874 | 2.1 | 92.8 | 6.5 |
| 17 | Procyanidin B2 | M+H | 9261 | 4.57 | C_30_H_26_O_12_ | 579.15 | 579.1493 | -0.7 | 75.8 | 2.9 |
| 18 | Syringin +NH_3_ | M+H | 4417 | 5.01 | C_17_H_24_O_9_.NH_3_ | 390.176 | 390.1758 | -0.2 | 82 | 16 |
| 19 | Chlorogenic acid | M+H | 1130000 | 5.03 | C_16_H_18_O_9_ | 355.102 | 355.102 | -0.9 | 97.8 | 6.2 |
| 20 | Vitamin B_2_ | M+H | 40890 | 5.83 | C_17_H_20_N_4_O_6_ | 377.146 | 377.1452 | -0.9 | 99.6 | 2.7 |
| 21 | Pinoresinol Diglucoside +NH_3_ | M+H | 7168 | 6.13 | C_32_H_42_O_16_.NH_3_ | 700.281 | 700.2805 | -0.9 | 92.7 | 1.2 |
| 22 | Schaftoside | M+H | 40320 | 6.25 | C_26_H_28_O_14_ | 565.155 | 565.1547 | -0.9 | 86.7 | 7.1 |
| 23 | E Eleutheroside E +NH_3_ | M+H | 7324 | 6.52 | C_34_H_46_O_18_.NH_3_ | 760.302 | 760.3023 | 0.1 | 73.2 | 8 |
| 24 | Quercetin | M+H | 251200 | 7.2 | C_15_H_10_O_7_ | 303.05 | 303.0494 | -1.6 | 82.9 | 4.9 |
| 25 | Hyperin | M+H | 354800 | 7.2 | C_21_H_20_O_12_ | 465.103 | 465.102 | -1.6 | 100 | 10 |
| 26 | Kaempferitrin | M+H | 46240 | 7.24 | C_27_H_30_O_14_ | 579.171 | 579.17 | -1.5 | 100 | 8.3 |
| 27 | Afzelin | M+H | 18560 | 7.24 | C_21_H_20_O_10_ | 433.113 | 433.1123 | -1.4 | 98.8 | 0.4 |
| 28 | Luteolin-7-O-β-D-glucuronide | M+H | 8237 | 7.42 | C_21_H_18_O_12_ | 463.087 | 463.0868 | -0.6 | 99.6 | 11 |
| 29 | Luteoloside | M+H | 95570 | 7.94 | C_21_H_20_O_11_ | 449.108 | 449.1075 | -0.8 | 100 | 8.3 |
| 30 | p-Anisic acid | M+H | 20310 | 9.06 | C_8_H_8_O_3_ | 153.055 | 153.0545 | -0.5 | 79 | 5.1 |
| 31 | Berberine | M+H | 4484 | 9.67 | C_20_H_17_NO_4_ | 336.123 | 336.1233 | 0.8 | 94.7 | 4.8 |
| 32 | Epimedin A | M+H | 3517000 | 9.96 | C_39_H_50_O_20_ | 839.297 | 839.2958 | -1.2 | 98 | 16.3 |
| 33 | Epimedin B | M+H | 5516000 | 10.1 | C_38_H_48_O_19_ | 809.286 | 809.285 | -1.6 | 98 | 14.9 |
| 34 | Epimedin C | M+H | 4570000 | 10.25 | C_39_H_50_O_19_ | 823.302 | 823.3 | -2.3 | 99.4 | 15.8 |
| 35 | Icarrin | M+H | 21700000 | 10.5 | C_33_H_40_O_15_ | 677.244 | 677.2426 | -2.1 | 99.1 | 13.2 |
| 36 | Pratensein-7-O-glucoside | M+H | 12780 | 10.9 | C_22_H_22_O_11_ | 463.123 | 463.1223 | -2.5 | 83.3 | 4.9 |
| 37 | Icaritin | M+H | 18240 | 10.94 | C_21_H_22_O_7_ | 387.144 | 387.1436 | -0.5 | 99.2 | 14.4 |
| 38 | Baohuoside I | M+H | 4734000 | 14.89 | C_27_H_30_O_10_ | 515.191 | 515.1903 | -1.6 | 100 | 11.7 |
