# Supplementary material for: Erxian herbal pair enhances bone formation in infected bone nonunion models and attenuates lipopolysaccharide-induced osteoblastinhibition by regulating miRNA-34a-5p
Source: Bioengineered. 2023 Jan 24;13(6):14339–56. doi: 10.1080/21655979.2022.2085388 (PMC9995130; doi:10.1080/21655979.2022.2085388)
Supplement: Supplemental Material [file KBIE_A_2085388_SM9510.zip › supplementary/supplementary table 2.docx]

Table S2 Characterization of chemical constituents of EPH by UPLC-Q/TOF-MS (Negative Mode).

| NO. | Component Name | Adduct | Area | Retention Time | Formula | Precursor Mass | Found At Mass | Mass Error (ppm) | Library Score | Isotope Ratio Difference |
| --- | --- | --- | --- | --- | --- | --- | --- | --- | --- | --- |
| 1 | L(+)-Arginine | M-H | 52270 | 1.07 | C_6_H_14_N_4_O_2_ | 173.104 | 173.1042 | -1.2 | 94.4 | 0.9 |
| 2 | Quinic acid | M-H | 9656000 | 1.25 | C_7_H_12_O_6_ | 191.056 | 191.0556 | -2.7 | 90.8 | 2.7 |
| 3 | L-Malic acid | M-H | 1219000 | 1.34 | C_4_H_6_O_5_ | 133.014 | 133.0141 | -0.9 | 78.5 | 0.7 |
| 4 | Adenine | M-H | 33900 | 1.62 | C_5_H_5_N_5_ | 134.047 | 134.047 | -1.5 | 96 | 1.9 |
| 5 | Citric acid | M-H | 857900 | 1.92 | C_6_H_8_O_7_ | 191.02 | 191.0193 | -2.1 | 98.8 | 2 |
| 6 | Succinic acid | M-H | 60350 | 2.36 | C_4_H_6_O_4_ | 117.019 | 117.0191 | -1.7 | 98 | 1.3 |
| 7 | Guanosine | M-H | 163700 | 2.59 | C_10_H_13_N_5_O_5_ | 282.084 | 282.0838 | -2 | 98.3 | 7.2 |
| 8 | Gallic acid | M-H | 126300 | 2.75 | C_7_H_6_O_5_ | 169.014 | 169.014 | -1.2 | 96.9 | 2.8 |
| 9 | Phenprobamate | M-H | 129700 | 3.36 | C_9_H_11_NO_2_ | 164.072 | 164.0714 | -1.9 | 97.8 | 4.4 |
| 10 | Higenamine | M-H | 2796 | 4.43 | C_16_H_17_NO_3_ | 270.114 | 270.1136 | 0 | 98.3 | 4.3 |
| 11 | L-Tryptophan | M-H | 118700 | 4.45 | C_11_H_12_N_2_O_2_ | 203.083 | 203.0822 | -1.8 | 94.7 | 5.7 |
| 12 | Salidroide | M-H | 25090 | 4.5 | C_14_H_20_O_7_ | 299.114 | 299.1131 | -1.6 | 87.4 | 6.2 |
| 13 | Esculin hydrate | M-H | 17900 | 4.51 | C_15_H_16_O_9_ | 339.072 | 339.0718 | -1.1 | 83.3 | 4.2 |
| 14 | Procyanidin B2 | M-H | 15080 | 4.56 | C_30_H_26_O_12_ | 577.135 | 577.1347 | -0.8 | 82.7 | 14.3 |
| 15 | Orcinol glucosid | M-H | 1634000 | 4.6 | C_13_H_18_O_7_ | 285.098 | 285.0977 | -1 | 98 | 6.1 |
| 16 | Orcinol glucosid +HCOOH | M-H | 3548000 | 4.6 | C_13_H_18_O_7_.HCOOH | 331.103 | 331.103 | -1.3 | 87.2 | 7.7 |
| 17 | Protocatechuic Aldehyde | M-H | 316000 | 4.93 | C_7_H_6_O_3_ | 137.024 | 137.024 | -2.8 | 96.7 | 2.6 |
| 18 | Chlorogenic acid | M-H | 3281000 | 5.03 | C_16_H_18_O_9_ | 353.088 | 353.087 | -2.2 | 99.3 | 6.8 |
| 19 | Catechin | M-H | 138400 | 5.1 | C_15_H_14_O_6_ | 289.072 | 289.0715 | -1.1 | 95.4 | 5.8 |
| 20 | Esculetin | M-H | 81040 | 5.53 | C_9_H_6_O_4_ | 177.019 | 177.0189 | -2.3 | 97.9 | 4.1 |
| 21 | Caffeic acid | M-H | 366800 | 5.63 | C_9_H_8_O_4_ | 179.035 | 179.0344 | -3.2 | 73.2 | 3.6 |
| 22 | Acteoside; Verbascoside; Kusaginin | M-H | 81280 | 6.11 | C_29_H_36_O_15_ | 623.198 | 623.1969 | -2 | 83.8 | 8 |
| 23 | Pinoresinol Diglucoside | M-H | 19010 | 6.13 | C_32_H_42_O_16_ | 681.24 | 681.2383 | -2.5 | 96.8 | 6.1 |
| 24 | Pinoresinol Diglucoside +HCOOH | M-H | 43370 | 6.13 | C_32_H_42_O_16_.HCOOH | 727.245 | 727.2438 | -2.3 | 92.5 | 15.2 |
| 25 | Isoschaftoside | M-H | 55590 | 6.24 | C_26_H_28_O_14_ | 563.141 | 563.1397 | -1.7 | 98.5 | 9.9 |
| 26 | Calceorioside B | M-H | 602700 | 6.28 | C_23_H_26_O_11_ | 477.14 | 477.1392 | -2.2 | 90 | 10.9 |
| 27 | Eleutheroside E +HCOOH | M-H | 46140 | 6.53 | C_34_H_46_O_18_.HCOOH | 787.267 | 787.2647 | -2.4 | 99.7 | 9.9 |
| 28 | p-Coumaric acid | M-H | 100300 | 6.83 | C_9_H_8_O_3_ | 163.04 | 163.0396 | -2.7 | 97.5 | 4.8 |
| 29 | Rutin | M-H | 10990 | 6.98 | C_27_H_30_O_16_ | 609.146 | 609.1445 | -2.7 | 98.4 | 9.8 |
| 30 | Hyperin | M-H | 1251000 | 7.19 | C_21_H_20_O_12_ | 463.088 | 463.0871 | -2.3 | 99.5 | 9.9 |
| 31 | Kaempferitrin | M-H | 84830 | 7.24 | C_27_H_30_O_14_ | 577.156 | 577.1552 | -1.9 | 97.1 | 11.8 |
| 32 | Scutellarin | M-H | 11870 | 7.41 | C_21_H_18_O_12_ | 461.073 | 461.0715 | -2.3 | 91.3 | 5.1 |
| 33 | 3,4-DCQA Isochlorogenic acid B | M-H | 43600 | 7.76 | C_25_H_24_O_12_ | 515.119 | 515.1183 | -2.4 | 98.3 | 5.1 |
| 34 | Astragalin | M-H | 441800 | 7.93 | C_21_H_20_O_11_ | 447.093 | 447.0921 | -2.5 | 97.7 | 11.1 |
| 35 | Pinoresinol-glucoside | M-H | 55570 | 7.94 | C_26_H_32_O_11_ | 519.187 | 519.1858 | -2.7 | 97.1 | 7.8 |
| 36 | Rosmarinic acid | M-H | 166800 | 8.55 | C_18_H_16_O_8_ | 359.077 | 359.0761 | -3.1 | 99.5 | 8.7 |
| 37 | Curculigoside | M-H | 932500 | 8.6 | C_22_H_26_O_11_ | 465.14 | 465.1391 | -2.5 | 89.7 | 7.7 |
| 38 | Curculigoside +HCOOH | M-H | 2683000 | 8.6 | C_22_H_26_O_11_.HCOOH | 511.146 | 511.1441 | -3.1 | 81.4 | 10.3 |
| 39 | Afzelin | M-H | 62660 | 8.8 | C_21_H_20_O_10_ | 431.098 | 431.097 | -3.1 | 98.4 | 8.6 |
| 40 | Phloridzin | M-H | 594400 | 8.81 | C_21_H_24_O_10_ | 435.13 | 435.1281 | -3.6 | 99.2 | 9 |
| 41 | Pseuoginsenoside F11 +HCOOH | M-H | 40890 | 9.78 | C_42_H_72_O_14_.HCOOH | 845.49 | 845.4877 | -3.2 | 93.4 | 17 |
| 42 | Epimedin A | M-H | 630100 | 9.96 | C_39_H_50_O_20_ | 837.282 | 837.2787 | -4.2 | 100 | 15 |
| 43 | Epimedin A +HCOOH | M-H | 2642000 | 9.97 | C_39_H_50_O_20_.HCOOH | 883.288 | 883.2837 | -4.6 | 81.2 | 8.1 |
| 44 | Quercetin | M-H | 7569 | 10.08 | C_15_H_10_O_7_ | 301.035 | 301.0344 | -3.3 | 78.9 | 2.9 |
| 45 | Epimedin B +HCOOH | M-H | 3820000 | 10.1 | C_38_H_48_O_19_.HCOOH | 853.277 | 853.2747 | -2.9 | 84.7 | 17.5 |
| 46 | Naringenin | M-H | 5720 | 11.3 | C_15_H_12_O_5_ | 271.061 | 271.0608 | -1.4 | 87.6 | 8.8 |
| 47 | Pedunculoside +HCOOH | M-H | 13800 | 11.56 | C_36_H_58_O_10_.HCOOH | 695.401 | 695.3984 | -4 | 95.9 | 5.5 |
| 48 | Ginsenoside Rd +HCOOH | M-H | 22730 | 12.05 | C_48_H_82_O_18_.HCOOH | 991.548 | 991.5447 | -3.7 | 93.7 | 13.1 |
| 49 | 20(S)Ginsenoside Rg3 +HCOOH | M-H | 116000 | 12.49 | C_42_H_72_O_13_.HCOOH | 829.495 | 829.4922 | -4 | 97.1 | 15.3 |
| 50 | Madecassic acid | M-H | 46630 | 12.84 | C_30_H_48_O_6_ | 503.338 | 503.3357 | -4.2 | 75.6 | 9 |
| 51 | Saikosaponin D +HCOOH | M-H | 9895 | 13.74 | C_42_H_68_O_13_.HCOOH | 825.464 | 825.4612 | -3.6 | 82.4 | 2.7 |
| 52 | Baohuoside I | M-H | 7225000 | 14.89 | C_27_H_30_O_10_ | 513.177 | 513.1749 | -3.3 | 97.4 | 10.9 |
| 53 | Linoleic acid | M-H | 2507 | 17.81 | C_18_H_32_O_2_ | 279.233 | 279.2321 | -3.1 | 80.9 | 1.8 |
