# Supplementary material for: Erxian herbal pair enhances bone formation in infected bone nonunion models and attenuates lipopolysaccharide-induced osteoblastinhibition by regulating miRNA-34a-5p
Source: Bioengineered. 2023 Jan 24;13(6):14339–56. doi: 10.1080/21655979.2022.2085388 (PMC9995130; doi:10.1080/21655979.2022.2085388)
Supplement: Supplemental Material [file KBIE_A_2085388_SM9510.zip › supplementary/supplementary table 3.docx]

Table S3 Characterization of chemical constituents of EPH.

| NO. | Component Name | Adduct | Ratioa (%) ^a^ | Retention Time | Formula |
| --- | --- | --- | --- | --- | --- |
| 1 | 20(S)Ginsenoside Rg3 +HCOOH | M-H | 0.15 | 12.49 | C_42_H_72_O_13_.HCOOH |
| 2 | 3,4-DCQA Isochlorogenic acid B | M-H | 0.06 | 7.76 | C_25_H_24_O_12_ |
| 3 | Acteoside; Verbascoside; Kusaginin | M-H | 0.11 | 6.11 | C_29_H_36_O_15_ |
| 4 | Adenine | M-H | 0.04 | 1.62 | C_5_H_5_N_5_ |
| 5 | Adenosine | M+H | 0.22 | 2.48 | C_10_H_13_N_5_O_4_ |
| 6 | Afzelin | M+H | 0.02 | 7.24 | C_21_H_20_O_10_ |
| 7 | Alanine | M+H | 0.04 | 1.11 | C_3_H_7_NO_2_ |
| 8 | Astragalin | M-H | 0.57 | 7.93 | C_21_H_20_O_11_ |
| 9 | Baohuoside I | M+H | 6.14 | 14.89 | C_27_H_30_O_10_ |
| 10 | Berberine | M+H | 0.01 | 9.67 | C_20_H_17_NO_4_ |
| 11 | Betaine | M+H | 0.51 | 1.16 | C_5_H_11_NO_2_ |
| 12 | Caffeic acid | M-H | 0.48 | 5.63 | C_9_H_8_O_4_ |
| 13 | Calceorioside B | M-H | 0.78 | 6.28 | C_23_H_26_O_11_ |
| 14 | Catechin | M-H | 0.18 | 5.1 | C_15_H_14_O_6_ |
| 15 | Chlorogenic acid | M+H | 1.47 | 5.03 | C_16_H_18_O_9_ |
| 16 | Citric acid | M-H | 1.11 | 1.92 | C_6_H_8_O_7_ |
| 17 | Curculigoside | M-H | 1.21 | 8.6 | C_22_H_26_O_11_ |
| 18 | Curculigoside +HCOOH | M-H | 3.48 | 8.6 | C_22_H_26_O_11_.HCOOH |
| 19 | Cytidine | M+H | 0.06 | 1.28 | C_9_H_13_N_3_O_5_ |
| 20 | E Eleutheroside E +NH_3_ | M+H | 0.01 | 6.52 | C_34_H_46_O_18_.NH_3_ |
| 21 | Eleutheroside E +HCOOH | M-H | 0.06 | 6.53 | C_34_H_46_O_18_.HCOOH |
| 22 | Epimedin A | M+H | 4.56 | 9.96 | C_39_H_50_O_20_ |
| 23 | Epimedin A +HCOOH | M-H | 3.43 | 9.97 | C_39_H_50_O_20_.HCOOH |
| 24 | Epimedin B | M+H | 7.16 | 10.1 | C_38_H_48_O_19_ |
| 25 | Epimedin B +HCOOH | M-H | 4.96 | 10.1 | C_38_H_48_O_19_.HCOOH |
| 26 | Epimedin C | M+H | 5.93 | 10.25 | C_39_H_50_O_19_ |
| 27 | Esculetin | M-H | 0.11 | 5.53 | C_9_H_6_O_4_ |
| 28 | Esculin hydrate | M+H | 0.01 | 4.52 | C_15_H_16_O_9_ |
| 29 | Gallic acid | M-H | 0.16 | 2.75 | C_7_H_6_O_5_ |
| 30 | Ginsenoside Rd +HCOOH | M-H | 0.03 | 12.05 | C_48_H_82_O_18_.HCOOH |
| 31 | Glutamic acid | M+H | 0.10 | 1.14 | C_5_H_9_NO_4_ |
| 32 | Guanosine | M+H | 0.13 | 2.61 | C_10_H_13_N_5_O_5_ |
| 33 | Higenamine | M+H | 0.04 | 4.44 | C_16_H_17_NO_3_ |
| 34 | Hyperin | M+H | 0.46 | 7.2 | C_21_H_20_O_12_ |
| 35 | Icaritin | M+H | 0.02 | 10.94 | C_21_H_22_O_7_ |
| 36 | Icarrin | M+H | 28.15 | 10.5 | C_33_H_40_O_15_ |
| 37 | Isoschaftoside | M-H | 0.07 | 6.24 | C_26_H_28_O_14_ |
| 38 | Kaempferitrin | M+H | 0.06 | 7.24 | C_27_H_30_O_14_ |
| 39 | L(+)-Arginine | M+H | 1.95 | 1.1 | C_6_H_14_N_4_O_2_ |
| 40 | L-Carnitine | M+H | 0.40 | 1.14 | C_7_H_15_NO_3_ |
| 41 | Linoleic acid | M-H | 0.00 | 17.81 | C_18_H_32_O_2_ |
| 42 | L-Malic acid | M-H | 1.58 | 1.34 | C_4_H_6_O_5_ |
| 43 | L-Tryptophan | M-H | 0.15 | 4.45 | C_11_H_12_N_2_O_2_ |
| 44 | Luteolin-7-O-β-D-glucuronide | M+H | 0.01 | 7.42 | C_21_H_18_O_12_ |
| 45 | Luteoloside | M+H | 0.12 | 7.94 | C_21_H_20_O_11_ |
| 46 | Madecassic acid | M-H | 0.06 | 12.84 | C_30_H_48_O_6_ |
| 47 | Naringenin | M-H | 0.01 | 11.3 | C_15_H_12_O_5_ |
| 48 | Nicotinamide | M+H | 0.12 | 1.88 | C_6_H_6_N_2_O |
| 49 | Nicotinic acid | M+H | 0.07 | 1.75 | C_6_H_5_NO_2_ |
| 50 | Orcinol glucosid | M-H | 2.12 | 4.6 | C_13_H_18_O_7_ |
| 51 | Orcinol glucosid +HCOOH | M-H | 4.60 | 4.6 | C_13_H_18_O_7_.HCOOH |
| 52 | p-Anisic acid | M+H | 0.03 | 9.06 | C_8_H_8_O_3_ |
| 53 | p-Coumaric acid | M-H | 0.13 | 6.83 | C_9_H_8_O_3_ |
| 54 | Pedunculoside +HCOOH | M-H | 0.02 | 11.56 | C_36_H_58_O_10_.HCOOH |
| 55 | Phenprobamate | M-H | 0.17 | 3.36 | C_9_H_11_NO_2_ |
| 56 | Phenylalanine | M+H | 0.55 | 3.35 | C_9_H_11_NO_2_ |
| 57 | Phloridzin | M-H | 0.77 | 8.81 | C_21_H_24_O_10_ |
| 58 | Pinoresinol Diglucoside | M-H | 0.02 | 6.13 | C_32_H_42_O_16_ |
| 59 | Pinoresinol Diglucoside +HCOOH | M-H | 0.06 | 6.13 | C_32_H_42_O_16_.HCOOH |
| 60 | Pinoresinol Diglucoside +NH_3_ | M+H | 0.01 | 6.13 | C_32_H_42_O_16_.NH_3_ |
| 61 | Pinoresinol-glucoside | M-H | 0.07 | 7.94 | C_26_H_32_O_11_ |
| 62 | Pratensein-7-O-glucoside | M+H | 0.02 | 10.9 | C_22_H_22_O_11_ |
| 63 | Procyanidin B2 | M+H | 0.01 | 4.57 | C_30_H_26_O_12_ |
| 64 | Proline | M+H | 0.43 | 1.23 | C_5_H_9_NO_2_ |
| 65 | Protocatechuic Aldehyde | M-H | 0.41 | 4.93 | C_7_H_6_O_3_ |
| 66 | Pseuoginsenoside F11 +HCOOH | M-H | 0.05 | 9.78 | C_42_H_72_O_14_.HCOOH |
| 67 | Quercetin | M+H | 0.33 | 7.2 | C_15_H_10_O_7_ |
| 68 | Quinic acid | M-H | 12.53 | 1.25 | C_7_H_12_O_6_ |
| 69 | Rosmarinic acid | M-H | 0.22 | 8.55 | C_18_H_16_O_8_ |
| 70 | Rutin | M-H | 0.01 | 6.98 | C_27_H_30_O_16_ |
| 71 | Saikosaponin D +HCOOH | M-H | 0.01 | 13.74 | C_42_H_68_O_13_.HCOOH |
| 72 | Salidroide | M-H | 0.03 | 4.5 | C_14_H_20_O_7_ |
| 73 | Salidroside +NH_3_ | M+H | 0.08 | 3.8 | C_14_H_20_O_7_.NH_3_ |
| 74 | Schaftoside | M+H | 0.05 | 6.25 | C_26_H_28_O_14_ |
| 75 | Scutellarin | M-H | 0.02 | 7.41 | C_21_H_18_O_12_ |
| 76 | Succinic acid | M-H | 0.08 | 2.36 | C_4_H_6_O_4_ |
| 77 | Syringin +NH_3_ | M+H | 0.01 | 5.01 | C_17_H_24_O_9_.NH_3_ |
| 78 | Trigonelline | M+H | 0.55 | 1.2 | C_7_H_7_NO_2_ |
| 79 | Vitamin B_2_ | M+H | 0.05 | 5.83 | C_17_H_20_N_4_O_6_ |

^a^ Ratio=Peak area of the analyte/total peak area of EHP × 100%
